# Opportunistic spawning of tropical anguillid eels *Anguilla bicolor bicolor* and *A. bengalensis bengalensis*

**DOI:** 10.1038/srep41649

**Published:** 2017-01-30

**Authors:** Takaomi Arai, Siti Raudah Abdul Kadir

**Affiliations:** 1 Environmental and Life Sciences Programme, Faculty of Science, Universiti Brunei Darussalam, Jalan Tungku Link, Gadong, BE 1410, Brunei Darussalam; 2 Institute of Oceanography and Environment, Universiti Malaysia Terengganu, 21030 Kuala Terengganu, Terengganu, Malaysia

## Abstract

Information on the spawning migration, spawning ecology and life history of tropical eels in the Indo-Pacific region is very limited. The physiological and morphological characteristics of tropical freshwater eels, *Anguilla bicolor bicolor* and *A. bengalensis bengalensis* collected in Malaysia were examined in relation to their downstream migration patterns. A total of 455 eels were collected over monthly intervals between February 2014 and January 2016 and we examined both gonadosomatic index and gonad histology features. In both species, close positive relationships between advanced maturation stages and eye, fin, gonadosomatic indexes were found in males and females. *A. bengalensis bengalensis* was found to be larger and heavier than *A. bicolor bicolor* at the time of seaward migration. The final stage of maturation for seaward spawning migration occurred throughout the year in *A. bicolor bicolor*, although that of *A. bengalensis bengalensis* was estimated to six months due to the limited number of samples. These results suggest that year-round spawning in the open ocean occurs in the tropical eel. This non-seasonal spawning ecology is notably different from that of temperate eels, which are known to follow a well-defined spawning season, with spawning migrations generally taking place during autumn months.

Being catadromous, the freshwater eel of the genus *Anguilla* migrates between fresh water growth habitats and offshore spawning areas. Nineteen species/subspecies of *Anguilla* have been found, 13 of which are known to live in tropical regions^1^,^2^. Of the latter, seven species/subspecies are found in the western Pacific around Indonesia and Malaysia, i.e., *A. celebesensis, A. interioris, A. bengalensis bengalensis, A. marmorata, A. borneensis, A. bicolor bicolor* and *A. bicolor pacifica*^1^,^2^,^3^,^4^. Molecular phylogenic research on freshwater eels has revealed that tropical eels are the most basal species originating from the Indonesian region and that freshwater eels radiate out from the tropics to colonize temperate regions^5^. Thus, studying the life history and migration patterns of tropical eels may provide clues to understanding the nature of primitive forms of catadromous migration in anguillid eels and how the migration patterns of this species were established. The results may also elucidate the evolutionary pathways of migration for this species and for other diadromous fish species that migrate between fresh water and seawater habitats.

The life cycle of the freshwater eel includes five stages: the leptocephalus, glass eel, elver, yellow eel and silver eel stages. Larvae, leptocephali, drift and are transported along ocean currents at the continental shelf. These leptocephali leave oceanic currents after metamorphosing into glass eels and then typically migrate upstream as elvers four to eight months after hatching^6^ to grow in fresh water habitats during the yellow stage (immature stage). After undergoing upstream migration, elvers become yellow eels and live in fresh water habitats such as rivers and lakes. Then, during the silver eel stage (early maturing stage) of the autumn and winter, their gonads begin to mature and they begin their downstream migration into the ocean and back out to the spawning area where they spawn and die.

Before migrating downstream to spawn, freshwater eels undergo a transition to prepare for oceanic life and in turn become silver eels^7^. The silver eel migration period is one of the life stages that is still poorly understood. The timing of downstream migration and its periodicity may impact survival rates, and such information may prove crucial to the development of successful protective measures^8^. Temperate eels are largely characterized by well-defined spawning and recruitment seasons, long larval durations, and panmictic populations. The spawning migration of temperate eels generally occurs during autumn months between August and December^9^,^10^,^11^ in the Northern Hemisphere and between February and May in the Southern Hemisphere^12^, except in a case of the prolonged downstream migration activity between April and December with high temporal variation in the European eel *Anguilla anguilla* in northeastern Germany^13^.

The tropical eel spawning period is still not well understood. Tropical eel recruitment periods differ depending on the species and/or sites involved^4^,^6^,^14^,^15^. *A. marmorata, A. bicolor bicolor* and *A. mossambica* have been found to be present for five months of the year on Réunion Island of the Mascarene Islands in the western Indian Ocean^14^. By contrast, *A. celebesensis, A. marmorata* and *A. bicolor pacifica* tropical eels exhibit year-round recruitment to their habitats in North Sulawesi, Indonesia in the western Pacific Ocean^4^,^6^,^15^. Recently, Arai *et al*.^16^ published initial traits of a tropical eel spawning period that extends throughout the year for *Anguilla bicolor bicolor* populations in Indonesia. These findings suggest that downstream migration and recruitment characteristics differ between temperate and tropical eels. The unique year-round spawning ecology and behaviours of tropical eels may facilitate mating between adult eels of different year classes (cohorts) or from different habitats. Such flexibility in the life history of tropical eels may help to maintain their populations relative to those of temperate eels. However, few studies have confirmed the timing and seasonality of seaward migration for spawning among tropical eel species.

The purpose of the present study was to understand the timing of maturation and downstream migration patterns among tropical freshwater eels *Anguilla bicolor bicolor* and *A. bengalensis bengalensis* collected from northwestern Peninsular Malaysia, Malaysia, using specimens collected monthly basis. The timing of maturation and downstream migration was examined by investigating fluctuations in both the gonadosomatic index and gonadal histology levels. Morphometric variables of silver and yellow (immature) tropical eels were also compared across different maturation stages to understand how morphologies change with physiological changes. These data provide new information on freshwater eels, as knowledge on the maturation and life history of tropical eels is currently very limited.

## Results

### Environmental factors

**Figure 1:**
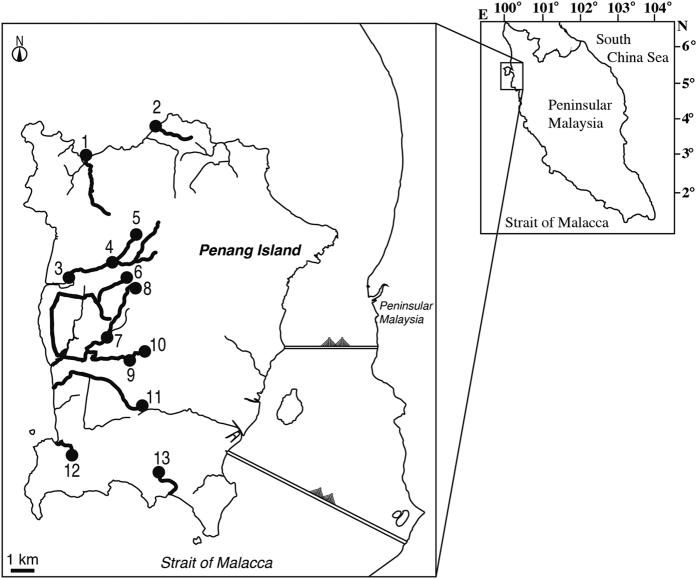
Locations where tropical freshwater eels were collected. Map showing the location of Penang Island in northwestern Peninsular Malaysia, Malaysia. The approximate locations where eels were collected from Penang Island are shown with closed circles. 1, Teluk Bahang in the downstream of the Teluk Bahang River, 2, Batu Ferringhi in the downstream of the Batu Ferringhi River, 3, Kuala Sungai Pinang in the downstream of the Pinang River, 4, Kampung Sungai Pinang in the midlestream of the Pinang River, 5, Titi Kerawang Waterfall in the upstream of the Pinang River, 6, Kampung Sungai Rusa in the midstream of the Rusa River, 7, Air Putih in the midstream of the Air Putih River, 8, Bandar Baru Air Putih in the upstream of the Air Putih River, 9, Titi Teras in the midstream of the Titi Teras River, 10, Titi Serong in the upstream of the Titi Teras River, 11, Pondok Upeh in the midstream of the Pondok Upeh River, 12, Pulau Betung in the midstream of the Pulau Betung River, 13, Bayan Lepas in the midstream of the Bayan Lepas River. The map was traced by the author used the Adobe Illustrator CS6 referring Google Maps 2016 (Map data ©2016 Google; https://maps.google.com/).

**Figure 2:**
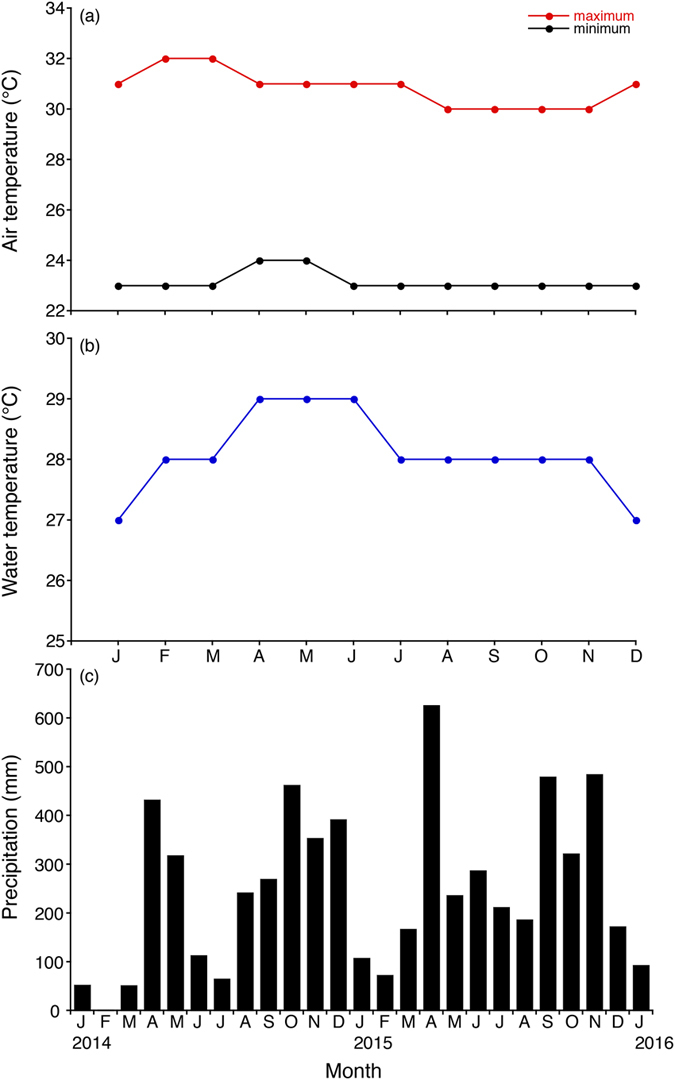
Environmental factors in the study area. Average monthly fluctuations in the air (**a**) and water temperatures (**b**) and the precipitation (**c**) in Penang Island in northwestern Peninsular Malaysia, Malaysia^17^,^18^.

Penang Island is an island of 293 km^2^ located in the Strait of Malacca (Fig. 1). The island has a tropical climate, specifically a tropical rainforest climate bordering on a tropical monsoon climate. The monthly mean minimum and maximum air temperatures at the weather station in the island ranged from 23 to 24 °C and from 30 to 32 °C, respectively (Fig. 2a). The monthly mean water temperature ranged from 27 to 29 °C at the weather station (Fig. 2b). Water temperatures were variable among sampling sites ranged from 24 to 36 °C. These results suggest that the temporal variations of water temperature are small in comparison to the spatial variability in the study area. High precipitation was observed throughout the year with peaks in April and October 2014 and April, September and November 2015 except in January and February for each year (Fig. 2c).

### Species and sex compositions

Eels were found to exist throughout the year, with *Anguilla bicolor bicolor* being the dominant species, constituting 87.9% of the eel sample, followed by *A. bengalensis bengalensis* (11.9%) and *A. marmotrata* (0.2%; one specimen), respectively. For *A. bicolor bicolor*, a total of 291 and 51 specimens were determined to be female and male, respectively, while the sexes of 58 of the 400 specimens could not determined due to the presence of undeveloped gonads (Table 1). A total of 40 and 7 *A. bengalensis bengalensis* specimens were determined to be female and male, respectively, and the sexes of 7 of the 54 specimens could not be determined due to the existence of undeveloped gonads (Table 1). One specimen of *A. marmorata* was determined to be female (Table 1). We used 389 *A. bicolor bicolor* (342) and *A. bengalensis bengalensis* (47) specimens with determined sexes for further analysis.

### Female GSI and histology

**Figure 3:**
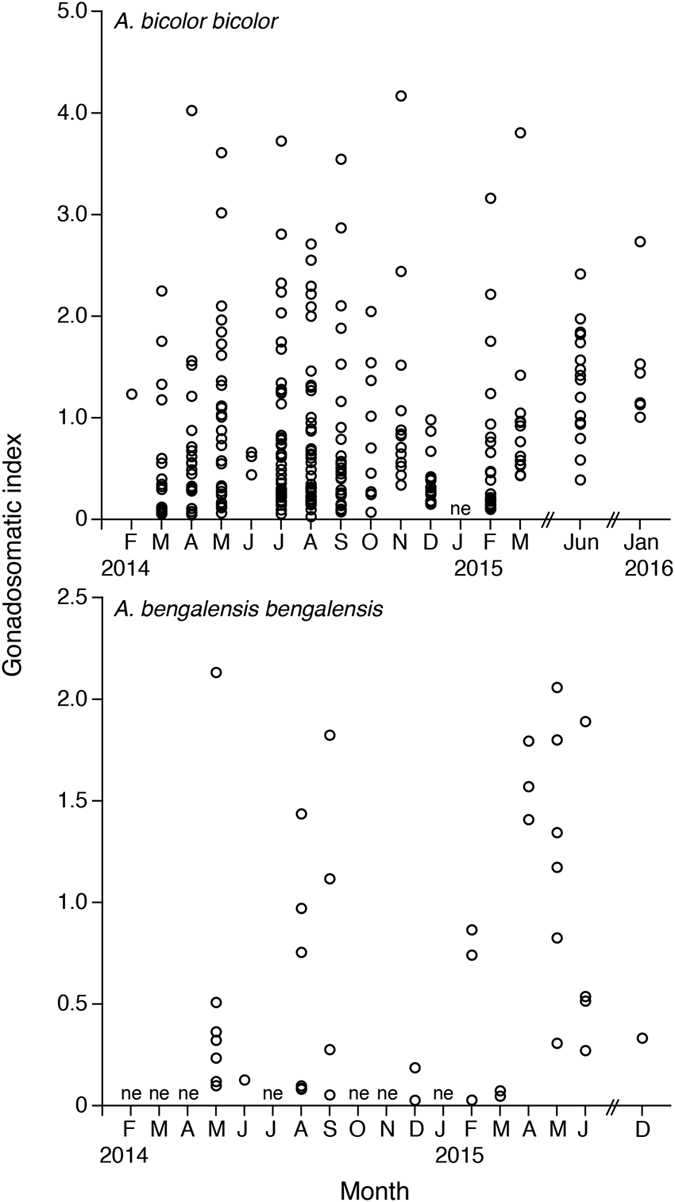
Reproductive characters of tropical freshwater eels. Monthly fluctuations in the gonadosomatic index for female tropical anguillid eels (*Anguilla bicolor bicolor* (top) and *A. bengalensis bengalensis* (bottom)) collected from Penang Island in northwestern Peninsular Malaysia, Malaysia between February 2014 and January 2016. ne: studies were conducted, but no eels were collected.

**Figure 4:**
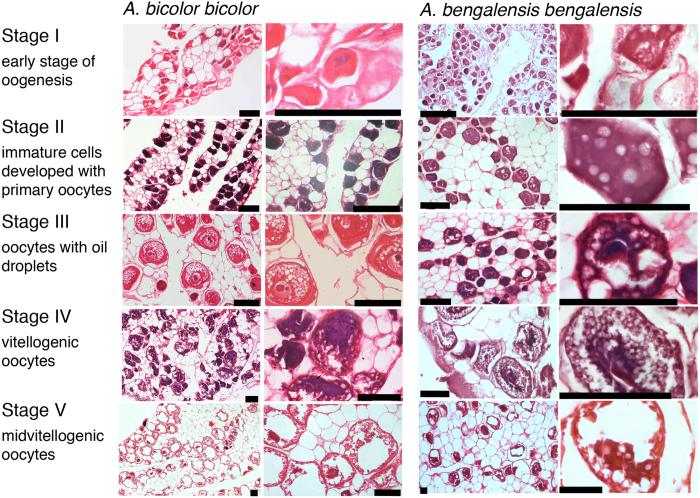
Gonadal development of tropical freshwater eels. Gonadal histology of *Anguilla bicolor bicolor* (left) and *A. bengalensis bengalensis* (right) female collected from Penang Island of northwestern Peninsular Malaysia, Malaysia between February 2014 and January 2016. Stages correspond to a growth phase (stages I and II), a pre-migrant phase (III) and two migrating phases (IV and V). Each stage was modified according to Lokman *et al*.^19^. Each scale bar is 100 μm.

**Figure 5:**
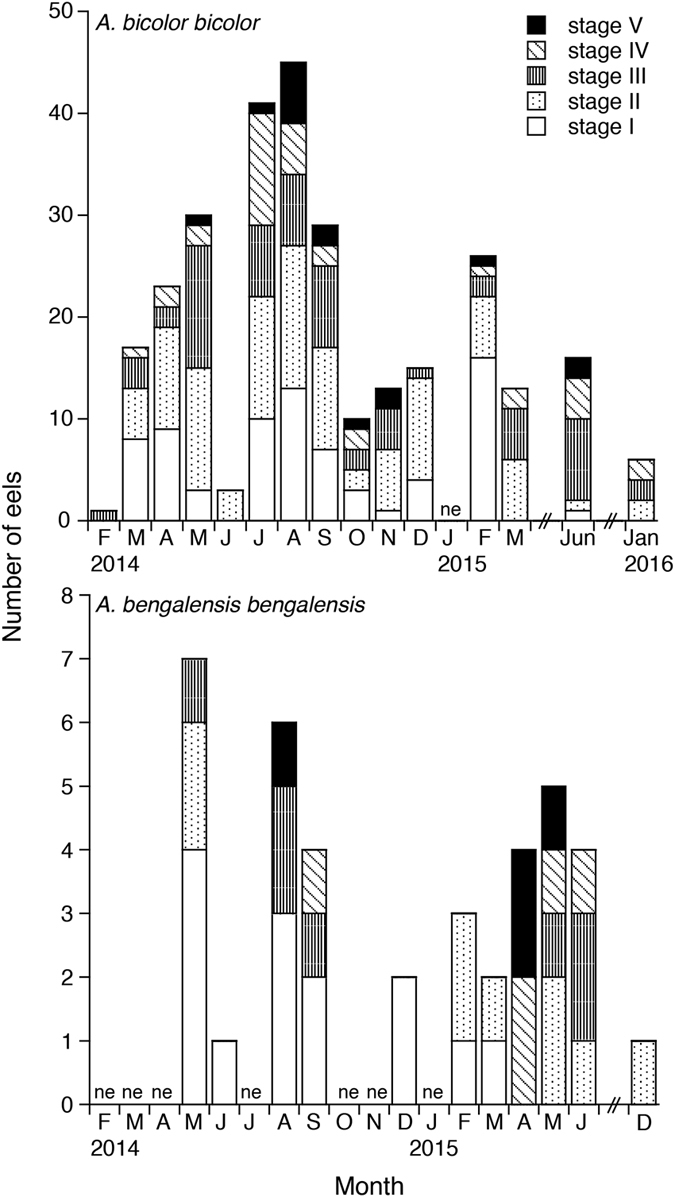
Reproductive characters of tropical freshwater eels. Monthly fluctuations in maturation stages of female tropical anguillid eels (*Anguilla bicolor bicolor* (top) and *A. bengalensis bengalensis* (bottom)) collected from Penang Island of northwestern Peninsular Malaysia, Malaysia between February 2014 and January 2016. ne: studies were conducted, but no eels were collected.

Female GSIs ranged from 0.03 to 4.17 in *Anguilla bicolor bicolor* and from 0.03 to 2.13 in *A. bengalensis bengalensis* (Table 1), and these values were highly variable between months for each species (Fig. 3). The gonadal histology of transverse sections was the same between the examined species, demonstrating that oocyte characteristics of each maturation stage also varied between months from Stage I (primary stage) to Stage V (final stage for spawning) (Fig. 4). In Stage I during early stages of oogenesis, gonads were composed of primary germ cells, oogonia, lamellae and primary oocytes. During Stage II, immature cells developed with primary oocytes and a few oil droplets. The early maturation stage of Stage III involved the development of oocytes with cortical alveoli and oil droplets in the cytoplasm (Fig. 4). The early vitellogenic stage of Stage IV involved vitellogenic oocyte, nucleus, yolk granule and central yolk platelet development and the oocyte diameter increased drastically while the cytoplasm filled with yolk granules (Fig. 4). During the final stage of maturation (Stage V), midvitellogenic oocytes formed in final preparation for spawning; disintegrated nuclei were observed and whole nuclei were no longer visible (Fig. 4). Eels occupying more developed stages (e.g., Stages IV and V) were found every month, although their numbers varied between months in the *A. bicolor bicolor* samples (Fig. 5). These findings suggest that *A. bicolor bicolor* undergo seaward migration for spawning throughout the year. In *A. bengalensis bengalensis*, IV and V maturation stages occurred together between April and September, although we did not find eels each month and we found fewer specimens compared to those of *A. bicolor bicolor* for examination (Fig. 5).

### Male GSI and histology

**Figure 6:**
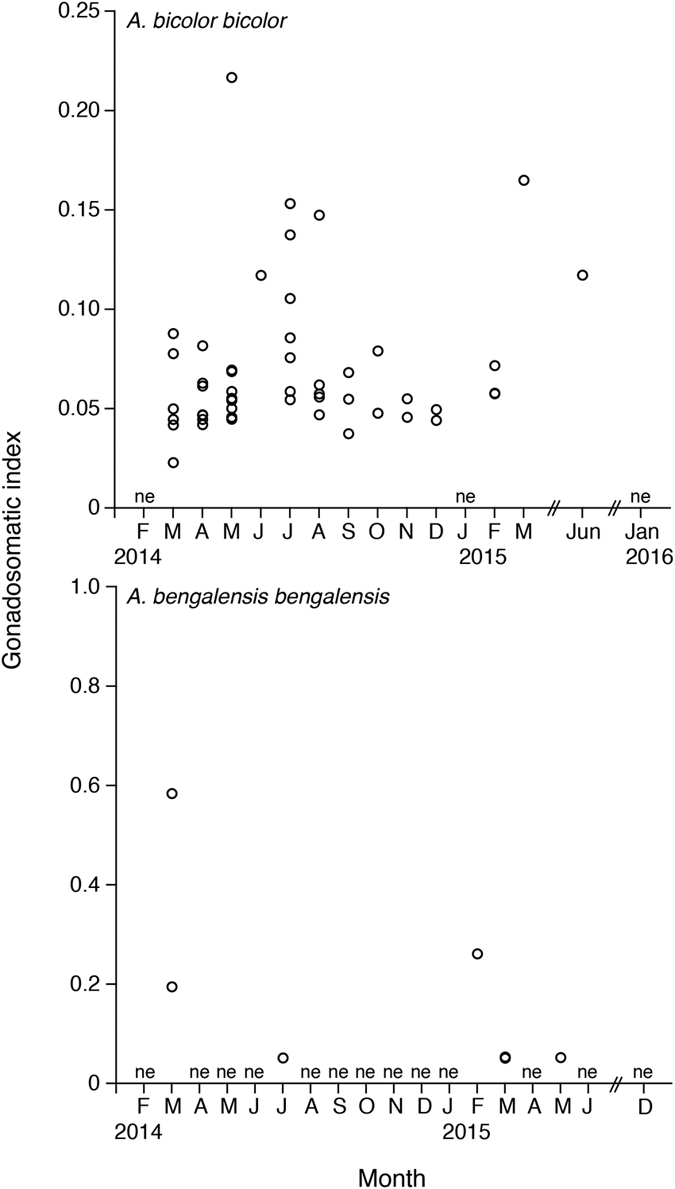
Reproductive characters of tropical freshwater eels. Monthly fluctuations in the gonadosomatic index of male in tropical anguillid eels (*Anguilla bicolor bicolor* (top) and *A. bengalensis bengalensis* (bottom)) collected from Penang Island of northwestern Peninsular Malaysia, Malaysia between February 2014 and January 2016. ne: studies were conducted, but no eels were collected.

**Figure 7:**
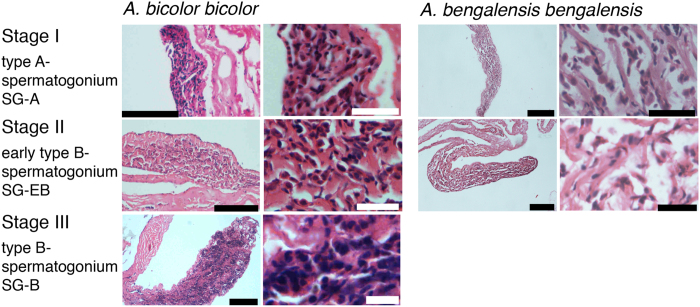
Gonadal development of tropical freshwater eels. Gonadal histology of *Anguilla bicolor bicolor* (left) and *A. bengalensis bengalensis* (right) males collected from Penang Island of northwestern Peninsular Malaysia, Malaysia between February 2014 and January 2016. Stages correspond to immature (stage I), early maturation (stage II) and mid-maturation (stage III) stages. Each stage was modified according to Lokman *et al*.^20^ and Walsh *et al*.^21^. Each scale bar is 100 μm.

**Figure 8:**
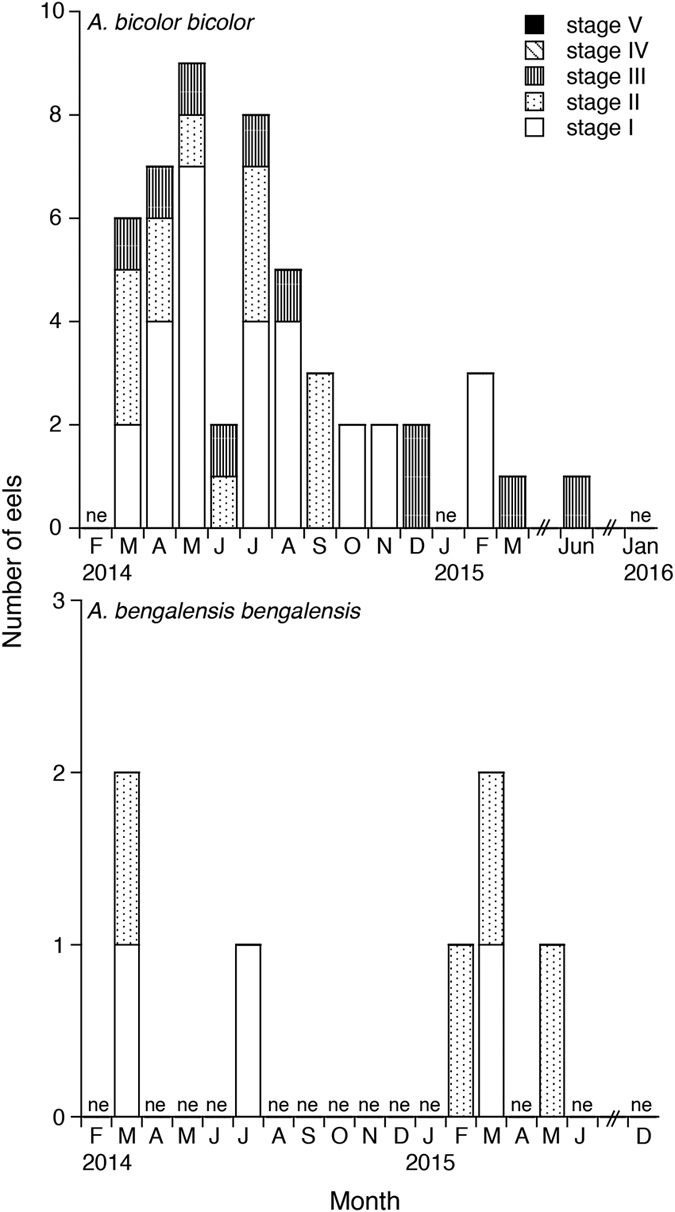
Reproductive characters of tropical freshwater eels. Monthly fluctuations in the maturation stages of male tropical anguillid eels (*Anguilla bicolor bicolor* (top) and *A. bengalensis bengalensis* (bottom)) collected from Penang Island of northwestern Peninsular Malaysia, Malaysia between February 2014 and January 2016. ne: studies were conducted, but no eels were collected.

Male GSIs ranged from 0.02 to 0.22 in *Anguilla bicolor bicolor* and from 0.05 to 0.58 in *A. bengalensis bengalensis* (Table 1), and values were variable between months for each species (Fig. 6). The gonadal histology of transverse sections was the same between species and revealed that oocyte characteristics of each maturation stage also varied within months from Stages I (primary stage) to III (middle maturation stage) in *A. bicolor bicolor* and from Stages I to Stage II (early maturation stage) in *A. bengalensis bengalensis* (Fig. 7). However, unlike the female eels, no male eels of either species reached the advanced maturation stages of Stages IV and V. During Stage I (immature testis stage), the development of seminiferous tubules showed a dominance of spermatogonia type A (SG-A) with early type B spermatogonia (SG-EB) in tubules with Leydig cells scattered between tubules. During Stage II, the size of seminiferous tubules increased and generated SG-EB, type B spermatogonia (SG-B) and a few SG-A. The early maturation stage of Stage III exhibited a dense collection of cells in tubules with SG-B and spematocytes (SC) along inner margins. Spermatocytes are recognizable by their smaller nuclear size and heavily stained chromatin material (Fig. 7). Eels occupying early development stages, such as Stage III were found between March and August and in December in *A. bicolor bicolor* (Fig. 8). These findings suggest that maturation periods of *A. bicolor bicolor* extend longer throughout the year.

### Relationships between maturation stage and maturation indexes in females

**Figure 9:**
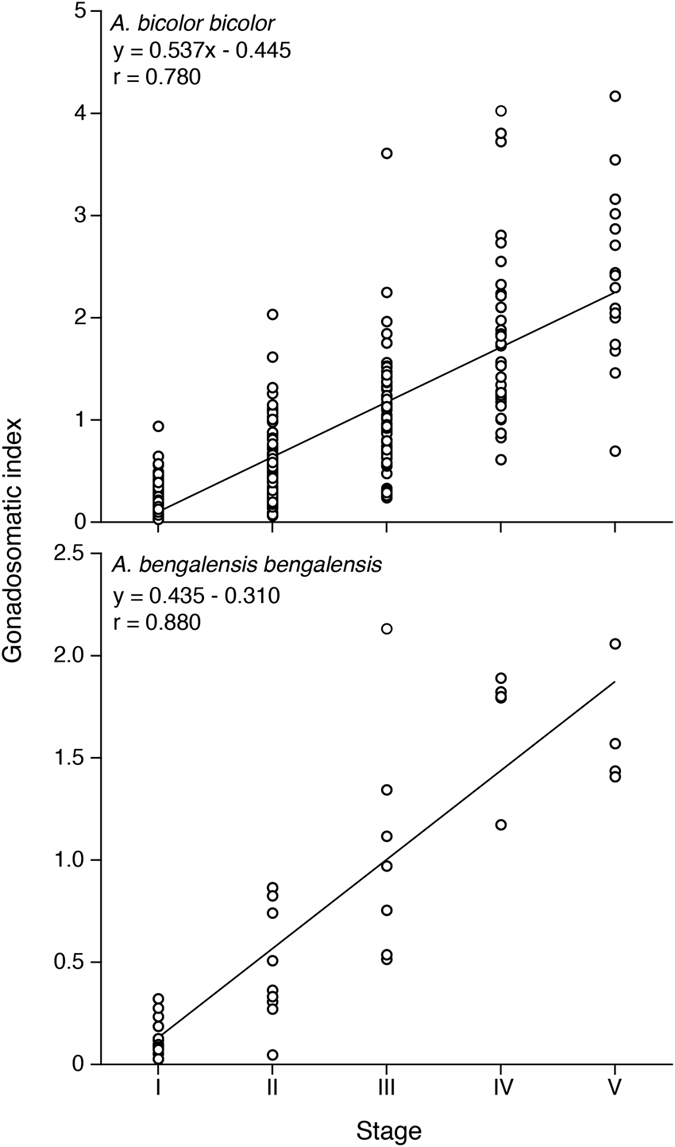
Reproductive characters of tropical freshwater eels. Relationships between maturation stages and gonadosomatic indexes of female tropical anguillid eels (*Anguilla bicolor bicolor* (top) and *A. bengalensis bengalensis* (bottom)) collected from Penang Island of northwestern Peninsular Malaysia, Malaysia between February 2014 and January 2016.

Mean female GSI values of each I, II, III, IV and V stage in *Anguilla bicolor bicolor* and *A. bengalensis bengalensis* were recorded as 0.23, 0.52, 1.05, 1.85 and 2.40 and as 0.13, 0.47, 1.05, 1.70 and 1.62, respectively (Table 1). Significant differences in GSI values between each stage were found for all combinations in both species (Fig. 9) (Kruskal-Wallis test, p < 0.05–0.0001) with the exception of combinations between Stages III and V and between Stages IV and V for *A. bengalensis bengalensis* (Kruskal-Wallis test, p > 0.05). Significantly positive correlations were found between maturation stages and GSI values (Fig. 9) (Fisher’s *Z*-Transformation, p < 0.0001). The GSI value of *A. bicolor bicolor* at the time of final maturation (Stage V) was significantly higher than that of *A. bengalensis bengalensis* (Mann Whitney *U*-test, p < 0.01).

**Figure 10:**
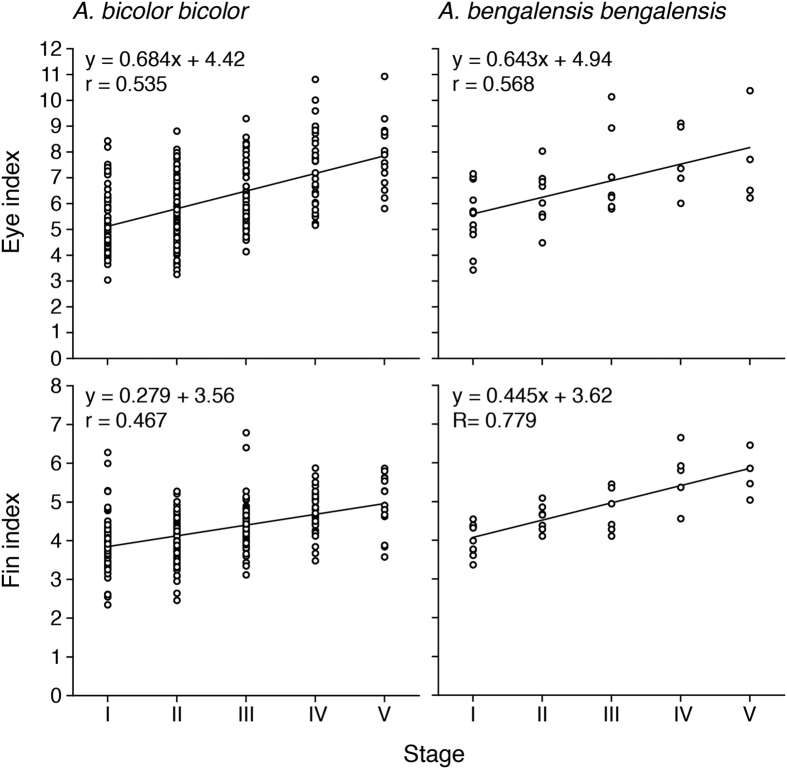
Reproductive characters of tropical freshwater eels. Relationships between maturation stages and eye (top) and fin indexes (bottom) of female tropical anguillid eels (*Anguilla bicolor bicolor* (left) and *A. bengalensis bengalensis* (right)) collected from Penang Island of northwestern Peninsular Malaysia, Malaysia between February 2014 and January 2016.

Mean EI and FL values found in female *Anguilla bicolor bicolor* for Stages I to V were recorded as 5.25, 5.68, 6.28, 7.44 and 7.92 and as 3.94, 3.98, 4.41, 4.79 and 4.93, respectively (Table 1). Those of female *A. bengalensis bengalensis* were recorded as 5.50, 6.19, 7.20, 7.69 and 7.71 and as 4.05, 4.58, 4.76, 5.67 and 5.71, respectively (Table 1). We found significant positive correlations between maturation stages and eye and fin indexes for both species (Fig. 10) (Fisher’s *Z*-Transformation, p < 0.0001). A significant positive correlation was found between the eye and fin indexes in both species (Fisher’s *Z*-Transformation, p < 0.0001).

### Relationships between maturation stages and TL and BW values in females

**Figure 11:**
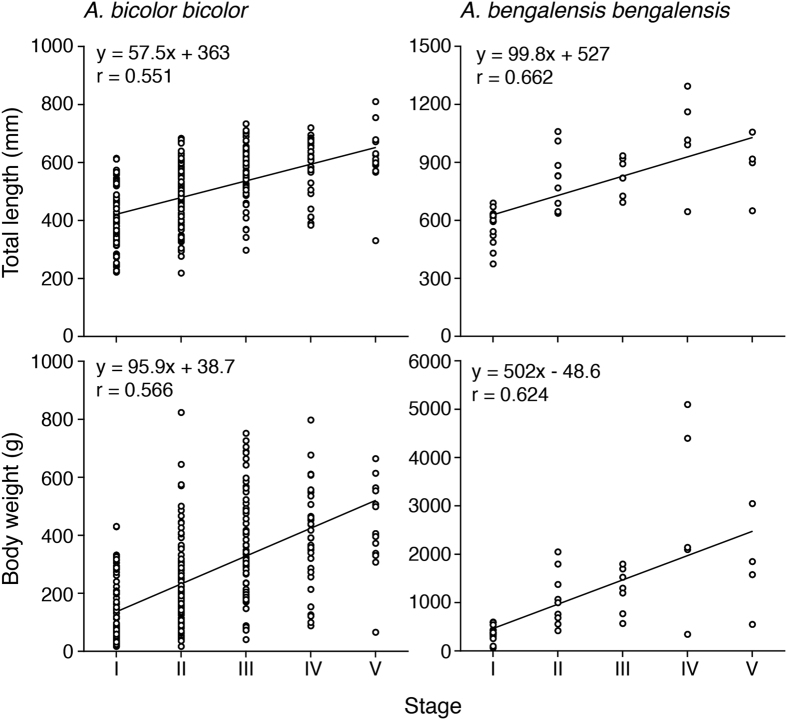
Reproductive characters of tropical freshwater eels. Relationships between maturation stages, total lengths (top) and body weights (bottom) of female tropical anguillid eels (*Anguilla bicolor bicolor* (left) and *A. bengalensis bengalensis* (right)) collected from Penang Island of northwestern Peninsular Malaysia, Malaysia between February 2014 and January 2016.

Mean TL and BW values in female *Anguilla bicolor bicolor* for Stages I to V were recorded as 411 mm, 475 mm, 565 mm, 580 mm and 615 mm and as 139 g, 216 g, 359 g, 391 g and 526 g, respectively (Table 1). Those of female *A. bengalensis bengalensis* were recorded as 573 mm, 818 mm, 830 mm, 1022 mm and 881 mm and as 365 g, 1081 g, 1267 g, 2818 g and 1757 g, respectively (Table 1). We found significant positive correlations between maturation stages and TL and BW values in both species (Fig. 11) (Fisher’s *Z*-Transformation, p < 0.0001). Significant differences in TL and BW values were found between *A. bicolor bicolor* and *A. bengalensis bengalensis* for each maturation stage from I to V (Kruskal-Wallis test, p < 0.05–0.0001). These results suggest that *A. bengalensis bengalensis* is larger and heavier than that of *A. bicolor bicolor* during each stage.

### Maturation and morphological characteristics of males

Mean male GSI values found for *Anguilla bicolor bicolor* during Stages I, II and III were 0.06, 0.07 and 0.09, respectively (Table 1). Those in *A. bengalensis bengalensis* recorded during Stages I and II were 0.10 and 0.30, respectively (Table 1). No significant differences in GSI values were found between any stages for both species (Kruskal-Wallis test, p > 0.05) unlike females.

**Figure 12:**
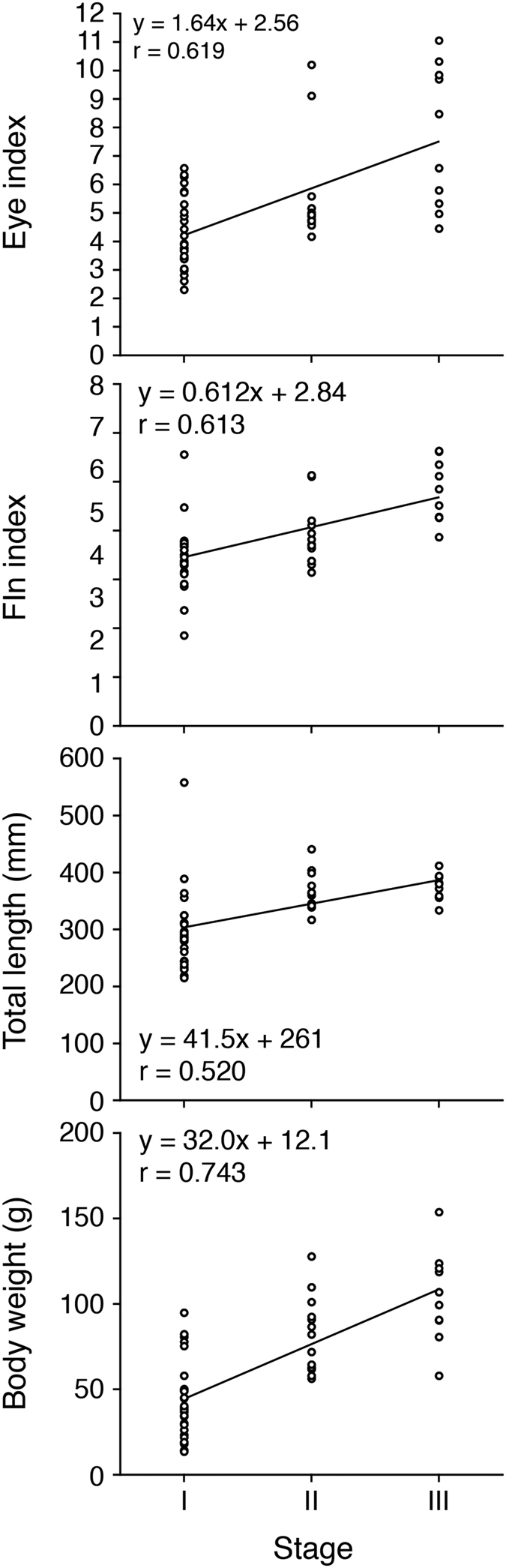
Reproductive characters of tropical freshwater eels. Relationships between maturation stages, eye (top) and fin indexes (upper middle), total lengths (lower middle) and body weights (bottom) of males tropical anguillid eels *Anguilla bicolor bicolor* collected from Penang Island of northwestern Peninsular Malaysia, Malaysia between February 2014 and January 2016.

Mean EI and FL values in male *Anguilla bicolor bicolor* for Stages I to III were recorded as 4.26, 5.59, 6.28 and 7.65 and as 3.49 and 3.94, 3.89 and 4.78, respectively (Table 1). Those of male *A. bengalensis bengalensis* for Stages I to II were recorded as 4.86 and 6.09 and as 4.73 and 4.52, respectively (Table 1). We found significantly positive correlations between maturation stages and eye and fin indexes in *A. bicolor bicolor* (Fig. 12) (Fisher’s *Z*-Transformation, p < 0.0001), although we could not conduct the same analyses of *A. bengalensis bengalensis* due to the occurrence of only two stages. A significant positive correlation was found between eye and fin indexes in *A. bicolor bicolor* (Fisher’s *Z*-Transformation, p < 0.0001), reflecting what was found for the females.

Mean TL and BW values in male *Anguilla bicolor bicolor* for Stages I to III were recorded as 298 mm, 362 mm and 374 mm and as 42.7 g, 82.0 g and 104 g, respectively (Table 1). Those of male *A. bengalensis bengalensis* for Stages I to Stage II were recorded as 422 mm and 480 mm and as 124 g and 151 g, respectively (Table 1). Significant positive correlations were found between maturation stages and TL and BW values in *A. bicolor bicolor* (Fig. 12) (Fisher’s *Z*-Transformation, p < 0.0001). Significant differences in TL and BW values were found between *A. bicolor bicolor* and *A. bengalensis bengalensis* during each maturation stage (I to II) (Kruskal-Wallis test, p < 0.0001). These results suggest that *A. bengalensis bengalensis* is larger and heavier *A. bicolor bicolor* during early maturation stages.

### Differences in TL and BW values between females and males

Significant differences in TL and BW values were found between females and males of *A. bicolor bicolor* (from Stage I to Stage III) and *A. bengalensis bengalensis* (from Stage I to Stage II) during each maturation stage (Kruskal-Wallis test, p < 0.05–0.0001). These results suggest that for both species, females are larger and heavier than males.

## Discussion

Due to the lack of existing studies on downstream migrating silver eels among tropical anguillid eels and due to difficulties associated with collecting migrating eels from coastal areas, information on migration levels and timing when such eels start spawning migration is scarce. In the present study, the spawning period of *Anguilla bicolor bicolor* females was found to extend throughout the year as revealed by our gonadal development and histology results (Figs 3 and 5). Although that of *A. bengalensis bengalensis* females was estimated at approximately 6 months based on the occurrence results found from monthly samples, mature eels were found in all of the months studied, and even in the limited samples collected compared to those of *A. bicolor bicolor* (Figs 3 and 5). Accordingly, we recommended that further studies are needed to elucidate whether the spawning season of *A. bengalensis bengalensis* females occurs throughout the year or restricted season. Recently, Arai *et al*.^16^ published an initial description of the spawning period of a tropical eel *A. bicolor bicolor* in Indonesia, which extends throughout the year. Thus, year-round spawning is a common characteristic of tropical anguillid eels. However, relative to the spawning migration of temperate eel species, considerably less research has examined the spawning migration of tropical eels. The findings described in this report indicate that tropical eels exhibit life history characteristics that differ markedly from those of temperate eels. Temperate anguillid species undergo spawning migration as silver eels during the fall and winter. Silver eels of the Japanese eel *Anguilla japonica*^22^,^23^, the American eel *A. rostrata*^11^, the European eel *A. anguilla*^7^,^24^,^25^,^26^, and the Australian and New Zealand eels *A. australis* and *A. dieffenbachii*^12^,^27^,^28^ descend through freshwater rivers and streams in the fall and enter saltwater, where they begin their marine migration to spawning areas in the open ocean. This difference in spawning season duration and timing between tropical and temperate species could be attributed to differences in the seaward migration seasons of maturing adult eels. Interestingly, GSIs and maturation stages were highly variable even within months for *A. bicolor bicolor* and *A. bengalensis bengalensis* (Figs 3 and 5). Such variations in GSIs and maturation stages found in tropical eels differ from those found for temperate eels characterized by a defined downstream season. Analyses of otolith microstructures show that the age of tropical eels at the time of recruitment remains constant throughout the year for *A. celebesensis, A. marmorata* and *A. bicolor pacifica*^6^. The year-round spawning migration of tropical species and constant larval growth extend the period of recruitment to estuarine habitats year-round for tropical eels. Spawning seasons of tropical eels have been found to extend throughout the year through a back calculation of otolith daily increments in *A. celebesensis, A. marmorata* and *A. bicolor pacifica* in North Sulawesi of Indonesia^6^, which is close to the area in Malaysia examined in the present study. In addition, the present results provide evidence of a year-round spawning period, as demonstrated by the monthly existence of matured eels.

Generally, the downstream migration of silver eels is considered as a seasonal phenomenon, which occurred in spring and between September and December each year in temperate eels^10^,^29^,^30^,^31^. Silvering is usually completed from September to November^32^. Recently, however, permanent monitoring in *Anguilla anguilla* in Warnow River, northeastern Germany found continuous migration activity between April and December with high temporal variation^13^. The silver eel migration peaks were also recorded in the summer or in winter with water temperatures less than 5 °C 13. The increased downstream migration activities during spring would be attributed to mature silver eels that stop their seaward movement at a specific point in the winter and wait for improving migration conditions in the next spring^10^. The observed prolonged downstream migration as well as the increased migration rates during the summer months might be related to the temporally discontinuous spawning migration of matured eels^33^. Therefore, silver eels might be able to detect beneficial downstream migration conditions and can conduct their seaward movement outside of the expected high migration periods^13^. Downstream migration activity of anguillid eels has been associated with numerous potential environmental predictors. These include hydrological variables (e.g. discharge, flow velocity and water temperature), climatic variables (e.g. barometric pressure, precipitation and air temperature), and the lunar cycle^7^,^13^,^29^,^30^,^31^. However, these triggers might not apply to *A. bicolor bicolor* and *A. bengalensis bengalensis* in Malaysia. Water discharge is quite different between the dry and wet season in Penang Island of Malaysia (Fig. 2c). However, downstream migration of *A. bicolor bicolor* was found to extend throughout the year (Fig. 5). Furthermore, air and water temperatures did not fluctuate dramatically throughout the year (Fig. 2a,b). This constant climate in Penang Island, Malaysia might induce non-seasonal seaward migration for spawning in tropical eels. The beginning and period of the downstream migration might be related to the geographical place of the continental life phase ensuring that seaward migrating eels arrive almost at the same time in the spawning area^34^. Therefore, further intensive studies should be undertaken in field regarding downstream migration in various localities and species in temperate eels as well as tropical eels to elucidate whether year-round downstream migration is species- and/or site-specific or common behavior in the anguillid eels.

This study is the first to describe the spawning period of tropical male eels, which extends throughout the year for *Anguilla bicolor bicolor* (Fig. 8). However, we could not determine this period for *A. bengalensis bengalensis* due to the limited number of samples examined. The spawning period of tropical male eels was also found to differ from that of temperate eels, which occurs from the autumn to the winter. These results suggest that the timing of downstream migration among tropical eels is same as that of female eels. The unique year-round spawning ecology and behaviour of tropical eels might facilitate mating between adult eels from different year classes (cohorts) or from different habitats. However, additional studies must be conducted to accumulate more information on male and female tropical eel spawning periods.

The degree of male gonadal developments was limited to Stage III of the middle maturation stage in *A. bicolor bicolor*. The limited testicular development of silver phase males in rivers was also found in the Japanese eel *A. japonica*^35^. These results indicate that males might be at premature stages in river environments before starting oceanic spawning migration and silver males might not have reached their maximum size and were still growing. Artificial propagation experiments indicated that the progress of spermatogenesis was faster than that of oogenesis after gonadotropin treatment^36^. Thus, to obtain simultaneous maturation between sexes, it would be necessary to initiate treatment of males later than females. These results suggest that gonadal developments of silver phase males might make more progress during their oceanic spawning migration than during their freshwater lives.

Sex determination in anguillid eels is believed to be controlled by environmental factors^37^,^38^. In the present study, female:male ratios of *Anguilla bicolor bicolor* and *A. bengalensis bengalensis* were commonly recorded as 85:15 for both species (Table 1). However, no *A. bicolor bicolor* males have been collected in Indonesia even though more than 400 specimens have been examined^16^. This difference, and the absence of males in populations in Indonesia under differing conditions among sites may influence eel sex determination results. Similar variations in sex ratios have been observed in *A. rostrata* by Oliveira and McCleave^39^, who reported that the sex ratios of silver eels differ in four rivers (males 49–77%) and that different factors influence sex determination in the rivers examined. For anguillid eels, it is believed that overcrowding and poor feeding give rise to development of males and that low population densities with rich feeding favour females^37^,^38^,^40^. This is supported by the fact that only males in some small streams exhibit strong recruitment patterns and high densities of young eels^41^.

The eels examined in this study show that *Anguilla bicolor bicolor* and *A. bengalensis bengalensis* females mature to a broad range of sizes and are larger and heavier than males occupying the same maturation stages (Table 1). This is similar for other species of anguillid eels, with females growing to much larger sizes than males^12^,^42^,^43^,^44^,^45^. These differences have been attributed to males eels using ‘a time-minimizing life history strategy’ to migrate at the earliest possible age and minimum size to mature and swim back to spawning areas, while females use ‘a time-maximizing strategy’ to migrate once their maximum body size has been achieved^46^.

A detailed examination of morphological and physiological characteristics reveals changes throughout tropical eel maturation. In both of the species examined, significant increases in EI, FI and GSI values in relation to downstream migration and maturation were observed (Figs 9,10,11 and 12). The same changes in relation to maturation have been found for temperate anguillid eels^7^,^47^,^48^. These changes appear to be ecologically and physiologically related to facilitating oceanic migrations. Eyes and pectoral fin growth occurs to meet the demands of deepwater swimming, predator avoidance and possibly visual mate location. It is highly probable that these changes constitute common characteristics of anguillid eels regardless of their growth habitats and migration distances for the onset of downstream migration to open seas through catadromous migration.

Degrees of gonadal development recorded at the time of downstream migration led by GSIs were found to overlap (1.70–2.40 on average and reaching maximum values of 2.06–4.17) between *Anguilla bicolor bicolor* and *A. bengalensis bengalensis* females in this study (Table 1). There is only limited available information on the maturation of tropical eels when they begin spawning migration. *A. celebesensis* GSI values collected from Poso Lake in Indonesia were found to be greater than 9.0 (11.2 in maximum value), falling within the range of GSI values characteristic of *A. japonica* and *A. marmorata* spawning conditions collected from offshore spawning grounds despite being collected inland from a freshwater lake far from the ocean^49^. *A. marmorata* GSI values collected from the same area were also found to be high; all GSI values were greater than 4.0 (6.4 in maximum value)^49^. However, clear differences in GSI values between *A. celebesensis* and *A. marmorata* for Poso Lake in Indonesia suggested that such maturation processes are related to how far spawning areas are from growth habitats^49^. *A. bicolor bicolor* GSI values collected from Indonesia were recorded as 2.5 on average, reaching a maximum value of 4.5 during Stage V 16 in accordance with the present results. Spawning areas of *A. celebesensis* are believed to be located in Tomini Bay of Sulawesi Island positioned < 100 kilometres from freshwater growth habitats^49^. This short spawning migration distance of <100 km is suggested induce the final stage of maturation in inland waters over a short period time for spawning areas to be reached^49^. However, the *A. marmorata* spawning area of the Poso population is suggested to be located far from Tomini Bay. The western North Pacific may thus serve as a possible spawning area from central Sulawesi, as these eels belong to the North Pacific population^49^. Compared to degrees of maturation among *A. celebesensis* and *A. marmorata* from Lake Poso, *A. bicolor bicolor* and *A. bengalensis bengalensis* spawning migration distances may be longer than those of eels occupying tropical waters.

In reference to tropical eels, Robinet *et al*.^14^ suggested a possible relationship between the advanced sexual maturation of *Anguilla bicolor bicolor* from Réunion Island (6.78 in GSI) and the previously estimated location of their spawning area (between 10 and 20 °S and 60–65 °E; Jubb, 1961). Furthermore, Arai^49^ reported a possible relationship between GSI values and spawning migration distances travelled by *A. celebesensis* and *A. marmorata*. Two temperate eels from New Zealand, *A. dieffenbachii* and *A. australis*, also show a difference in GSI values at the beginning of their spawning migration period, with females of the former species having a GSI of 8.1 and with the latter generating a GSI of 3.5 12. Jellyman^28^ suggests that this difference in sexual maturation may indicate that *A. dieffenbachii* travels a shorter distance to spawning areas than *A. australis* from their overlapping freshwater growth habitats. Recent results of a pop-up tag study, however, suggest that *A. dieffenbachii* is not a short distance migratory species and appears to spawn in the southern Fiji basin^50^, though this may still be a shorter migration route than that of *A. australis*, which appears to spawn further north in the South Equatorial Current^51^. Rates of *A. anguilla* and *A. japonica* silver eel maturation at the start of the spawning migration period may be reflected by differences in migration route distances relative to spawning areas (*A. anguilla*: approximately 5000–8000 km; *A. japonica*: approximately 1000–3000 km).

Downstream migrating female European eels typically have GSI values of >1.2 24,25 but that are lower than 3.0 25,52. Female maturation among Japanese eels as they began their spawning migration in coastal areas was found to range from 1.0 to 4.0 in GSI^9^. In the present study, GSI values of *Anguilla bicolor bicolor* and *A. bengalensis bengalensis* increased with advancing maturation stages (Fig. 9). These values were found to range from 0.7 to 4.2 for the former and from 1.4 to 2.1 for the latter at the maximum for Stage V (Table 1), and these values are overlap or are similar to those of *A. japonica* but are higher than those of *A. anguilla*. These results indicate that distances between freshwater growth habitats in Malaysia and *Anguilla bicolor bicolor* and *A. bengalensis bengalensis* spawning areas may span 1000–3000 km like that of *A. japonica*, although spawning areas in the Indian Ocean have not been identified yet.

The rate of maturation for anguillid eels at the onset of their seaward spawning migration may also reflect factors other than migration distances. For example, the TL values for the migration of female *Anguilla dieffenbachii* (950–1580 mm)^53^ and *A. marmorata* (832–1368 mm)^49^ are larger than the size of other anguillid eels. In the present study, the TL value at the time of spawning migration at Stage V for *A. bengalensis bengalensis* was found to be significantly larger than that of *A. bicolor bicolor* (Table 1). It is possible that this larger size permits higher swimming speeds that may reduce migration periods. The rate of maturation can also be affected by environmental or physiological factors such as temperature^54^,^55^, pressure^56^,^57^ and swimming habits^58^,^59^. Habitat environments and physiological factors should be the same between *A. bicolor bicolor* and *A. bengalensis bengalensis*, as these variables were measured for the same area. However, GSIs for the final stage of downstream migration (Stage V) for *A. bicolor bicolor* were significantly higher than those of *A. bengalensis bengalensis*. This difference may cause differences in swimming speed and in spawning areas from growth habitats.

Freshwater eel spawning areas are located in southern regions. Tropical eel spawning seasons were found to extend throughout the year in the present study and in previous studies^6^,^16^. The year-round spawning of tropical species and constant larval growth throughout the year^6^ extend the period of recruitment in estuarine habitats to last all year for tropical eels^4^,^15^. The present study and previous studies suggest that spawning migration scales are highly variable among tropical eel species. *Anguilla clelebesensis* and *A. borneensis* are categorized as small-scale migratory species (<100 km). *A. marmorata, A. bicolor bicolor* and *A. bengalensis bengalensis* are mid-scale migratory species (1000–3000 km) like the temperate eel *A. japonica*. However, their migration scales are smaller than those of other temperate species such as *A. rostrata* (1000–5000 km; mid- to large-scale migratory species) and *A. anguilla* (5000–8000 km; large-scale migratory species). Tropical eels are the most basal species originating from Indonesian and Malaysian regions, and these freshwater eels radiate out from the tropics to colonize temperate regions^5^. Tropical freshwater eels must be more closely related to the ancestral form than their temperate counterparts. Therefore, the origins of freshwater eel migration may be small in scale and reflect year-round spawning migration by tropical eels. During the dispersal and radiation process of oceanic migration worldwide, spawning migration scales can change gradually. For temperate eels, the retention of spawning areas in the tropics requires that eels migrate thousands of kilometres to follow clearly seasonal patterns of downstream migration and spawning in the open ocean.

## Methods

### Eels

A total of 455 anguillid eels were collected from Penang Island of the northwestern Peninsular Malaysia, Malaysia (approximately 5°21′–6°16′ N, 100°11′−102°42′E) (Fig. 1). The eels were collected using hook-and-line and eel traps at night in thirteen sites from nine rivers (Fig. 1). These rivers were Batu Ferringhi River, Teluk Bahang River, Pinang River, Rusa River, Air Putih River, Titi Teras River, Pondok Upeh River, Pulau Betung River, and Bayan Lepas River (Fig. 1). Pinang River areas were included downstream (Kuala Sungai Pinang), midstream (Kampung Sungai Pinang) and upstream (Titi Kerawang Waterfall) of Pinang River and Rusa River (midstream) (four locations) (Fig. 1). Air Putih and Titi Teras rives areas were included Bandar Baru Air Putih (upstream) and Air Putih (midstream) in the Air Putih River and Titi Serong (upstream) and Titi Teras (midstream) (four locations) (Fig. 1). Pondok Upeh is located on the midstream in the Pondok Upeh River. Sampling sites of Batu Ferringhi and Teluk Bahang are located in the downstream of the each river and those of Pulau Betung and Bayan Lepas River were located in the midstream areas (Fig. 1). A total of 4 locations, Batu Ferringhi, Teluk Bahang, Kuala Sg. Pinang and Pulau Betung, were influenced rising tide, while those of 9 locations, TitiKerawang Waterfall, Kampung Sungai Pinang, Kampung Sungai Rusa, Bandar Baru Air Putih, Air Putih, Titi Serong, Titi Teras, Pondok Upeh and Bayan Lepas were not influenced the effect. Water temperature and salinity levels were measured sporadically and ranged from 23.6 (upstream area) to 35.7 °C (downstream area) and from 0.01 (upstream area) to 32.9 ppt (downstream area), respectively. We also surveyed average monthly environmental parameters such as air temperature, water temperature and precipitation from data sources^17^,^18^. No specific permissions were required for these locations/activities, as the eel species involved is not endangered or protected and the collection area did not require permits to collect these animals. Our protocol was in accordance with a guide for animal experimentation at Universiti Malaysia Terengganu (UMT) and fish-handling approval was granted by the animal experiment committee of UMT. To examine temporal maturation variability levels, *Anguilla bicolor bicolor* specimens were collected on a monthly basis between February 2014 and March 2015 and those of *A. bengalensis bengalensis* were collected on a monthly basis between February 2014 and June 2015 (Fig. 1). Additional *A. bicolor bicolor* samples were collected in June 2015 and January 2016 and additional *A. bengalensis bengalensis* samples were collected in December 2015 to supplement samples collected monthly between February 2014 and March 2015. All eels were purchased post-mortem from local fishermen.

### Sample preparation

After the eels were collected, biological parameters (e.g., total length (TL) and body weight (BW)) were measured. Horizontal (E_h_, mm) and vertical (E_v_, mm) eye diameters and pectoral fin lengths (FL, mm) were measured to the nearest 0.01 mm using a digital caliper. Thereafter, the eye index (EI) was calculated^47^, EI = 100 πTL^−1^[0.25(E_h_ + E_v_)]^2^, and the pectoral fin length index (Fin index, FI) was also calculated, FI = 100 FLTL^−1^.

The sex of each eel was determined through visual and histological observations of gonads. The total gonad weight was measured, and the gonadosomatic index (GSI; percent relative gonad weight to BW) was subsequently calculated.

Anguillid eels found on Penang Island were identified through a morphological analysis, and they were further validated as *Anguilla bicolor bicolor, A. bengalensis bengalensis* and *A. marmorata* through an analysis of the eels’ mitochondrial cytochrome oxidase subunit I (COI) sequences and 16 S ribosomal RNA (16 S rRNA) according to Arai *et al*.^60^ and Arai and Wong^61^. A total of 400, 54 and 1 specimens were identified as *A. bicolor bicolor, A. bengalensis bengalensis* and *A. marmorata*, respectively.

### Histology analysis

All specimens were examined based on their histology. Fragments from the central region of one gonad were fixed in formalin for histological analysis. Tissue fragments were prepared for resin and routine paraffin embedding. Resin blocks were processed routinely and paraffin blocks were sectioned at 5 μm and were then stained with haematoxylin-eosin for histological observation. Histology classifications for females and males were carried out following Lokman *et al*.^19^, Lokman *et al*.^20^ and Walsh *et al*.^21^ with modifications.

### Statistical analysis

The differences in the GSI, EI, FI, TL and BW differences across stages for the examined species and between sexes were examined through a Kruskal-Wallis test. Consequently, post hoc Mann-Whitney-*U* tests were employed for between-species comparisons. Differences in GSI values between species at the final maturation stage (Stage V) were examined through a Mann-Whitney-*U* test. The significance of the correlation coefficient and regression slope was tested using Fisher’s Z-transformation^62^.

## Additional Information

**How to cite this article**: Arai, T. and Abdul Kadir, S. R. Opportunistic spawning of tropical anguillid eels *Anguilla bicolor bicolor* and *A. bengalensis bengalensis. Sci. Rep.* **7**, 41649; doi: 10.1038/srep41649 (2017).

**Publisher's note:** Springer Nature remains neutral with regard to jurisdictional claims in published maps and institutional affiliations.

## Figures and Tables

**Table 1:** Morphological characteristics of specimens used in the present study.

Species	Stage	Sample	Total length (mm)		Body weight (g)		Gonadosomatic index		Eye index		Fin index	
		size	mean ± SD	range	mean ± SD	range	mean ± SD	range	mean ± SD	range	mean ± SD	range
Female												
*A. bicolor bicolor*	I	78	411 ± 103	222–615	139 ± 107	17.3–431	0.23 ± 0.16	0.03–0.94	5.25 ± 1.13	3.05–8.44	3.94 ± 0.66	2.35–6.28
	II	99	475 ± 102	219–684	216 ± 148	17.5–824	0.52 ± 0.34	0.06–2.03	5.68 ± 1.26	3.26–8.81	3.98 ± 0.56	2.46–5.28
	III	64	565 ± 93.3	298–734	359 ± 179	41.2–752	1.05 ± 0.55	0.24–3.61	6.28 ± 1.23	4.15–9.30	4.41 ± 0.61	3.12–6.79
	IV	34	580 ± 94.7	384–720	391 ± 177	88.3–798	1.85 ± 0.84	0.62–4.03	7.44 ± 1.46	5.17–10.8	4.79 ± 0.56	3.49–5.88
	V	16	615 ± 101	331–810	526 ± 299	66.1–1267	2.40 ± 0.85	0.70–4.17	7.92 ± 1.29	5.81–10.9	4.93 ± 0.71	3.59–5.87
*A. bengalensis bengalensis*	I	14	573 ± 90.7	375–690	365 ± 158	65.8–600	0.13 ± 0.09	0.03–0.32	5.50 ± 1.12	3.43–7.16	4.05 ± 0.40	3.37–4.55
	II	9	818 ± 151	637–1060	1081 ± 561	421–2050	0.47 ± 0.28	0.05–0.87	6.19 ± 1.05	4.49–8.04	4.58 ± 0.34	4.11–5.09
	III	7	830 ± 93.7	694–935	1267 ± 462	568–1800	1.05 ± 0.56	0.52–2.13	7.20 ± 1.69	5.80–10.1	4.76 ± 0.57	4.11–5.45
	IV	5	1022 ± 244	645–1295	2818 ± 1923	345–5100	1.70 ± 0.29	1.17–1.89	7.69 ± 1.33	6.01–9.12	5.67 ± 0.77	4.56–6.66
	V	4	881 ± 169	650–1057	1757 ± 1027	550–3050	1.62 ± 0.30	1.41–2.06	7.71 ± 1.89	6.23–10.4	5.71 ± 0.60	5.04–6.46
*A. marmorata*	IV	1	904		2335		2.64		6.55		4.54	
Male												
*A. bicolor bicolor*	I	28	298 ± 67.1	215–558	42.7 ± 22.3	13.6–94.9	0.06 ± 0.03	0.04–0.22	4.26 ± 1.22	2.31–6.57	3.49 ± 0.64	1.85–5.56
	II	13	362 ± 35.5	317–441	82.0 ± 22.1	56.3–128	0.07 ± 0.04	0.02–0.15	5.59 ± 1.85	4.17–10.2	3.89 ± 0.63	3.14–5.14
	III	10	374 ± 22.9	334–412	104 ± 26.7	58.0–154	0.09 ± 0.04	0.04–0.17	7.65 ± 2.49	4.45–11.1	4.78 ± 0.63	3.87–5.63
*A. bengalensis bengalensis*	I	3	422 ± 57.0	357–462	124 ± 64.7	51.8–177	0.10 ± 0.08	0.05–0.20	4.86 ± 0.82	4.51–5.80	4.73 ± 0.97	4.04–5.41
	II	4	480 ± 21.2	464–557	151 ± 81.7	56.7–313	0.30 ± 0.27	0.05–0.58	6.09 ± 1.08	5.12–7.48	4.52 ± 0.05	4.48–4.66

A total of 58 and 7 specimens of *A. bicolor bicolor* and *A. bengalensis bengalensis*, respectively, are excluded due to undeveloped gonads and because sex could not be determined.
